# A Machine Learning Approach to Automated Structural Network Analysis: Application to Neonatal Encephalopathy

**DOI:** 10.1371/journal.pone.0078824

**Published:** 2013-11-25

**Authors:** Etay Ziv, Olga Tymofiyeva, Donna M. Ferriero, A. James Barkovich, Chris P. Hess, Duan Xu

**Affiliations:** 1 Department of Radiology & Biomedical Imaging, University of California San Francisco, San Francisco, California, United States of America; 2 Department of Pediatrics, University of California San Francisco, San Francisco, California, United States of America; Beijing Normal University, Beijing, China

## Abstract

Neonatal encephalopathy represents a heterogeneous group of conditions associated with life-long developmental disabilities and neurological deficits. Clinical measures and current anatomic brain imaging remain inadequate predictors of outcome in children with neonatal encephalopathy. Some studies have suggested that brain development and, therefore, brain connectivity may be altered in the subgroup of patients who subsequently go on to develop clinically significant neurological abnormalities. Large-scale structural brain connectivity networks constructed using diffusion tractography have been posited to reflect organizational differences in white matter architecture at the mesoscale, and thus offer a unique tool for characterizing brain development in patients with neonatal encephalopathy. In this manuscript we use diffusion tractography to construct structural networks for a cohort of patients with neonatal encephalopathy. We systematically map these networks to a high-dimensional space and then apply standard machine learning algorithms to predict neurological outcome in the cohort. Using nested cross-validation we demonstrate high prediction accuracy that is both statistically significant and robust over a broad range of thresholds. Our algorithm offers a novel tool to evaluate neonates at risk for developing neurological deficit. The described approach can be applied to any brain pathology that affects structural connectivity.

## Background

The term neonatal encephalopathy encompasses a heterogeneous group of conditions associated with life-long developmental disabilities and neurological deficits. It is an important clinical entity with prevalence ranging from 2 to 5 per 1000 live births [1]. Of these, up to 25% will go on to develop significant neurological deficits [2]. Some of these patients will have structural abnormalities evident on standard current imaging techniques [3]–[5], but the presence of anatomic lesions is an inadequate predictor of long-term outcome. A further complication is that some neonates may show delayed or transient abnormalities that may not be evident in the newborn period [6]. Early clinical measures generally lack sensitivity and specificity; more importantly, after the initial encephalopathy, symptoms may not become clinically manifest until the time window for intervention has passed. While the mechanism of injury in neonatal encephalopathy is multifactorial and nonuniform across patients [2], it has been hypothesized that the outcome results from alteration of the topology or connectivity of the developing brain [7]. The scale and heterogeneity of putative disruptions in normal network development and/or the emergence of altered connectivity patterns remains to be determined.

Recently there has been much interest in modeling brain connectivity as a network – defined as a set of nodes (representing brain regions) and connections between nodes (representing physical, correlational or functional interconnections between brain regions). To analyze the structure of these networks, researchers have borrowed and applied tools developed in the field of network theory. Typically these tools are graph-theoretic properties or statistics that can be quantified on real-world networks. Examples include clustering coefficient, mean path length, degree distribution, betweenness, modularity, and motifs [8]–[13]. Inherent to these approaches is the concept that statistics measured on real-world networks are significantly different than random networks, and this inherent difference in topology reveals an underlying design principle of the network.

In a *hypothesis-driven approach*, one may ask, for example, whether network metrics such as clustering coefficient or average path length correlate with neurological outcome. By contrast, in a *data-driven approach*, one can exploit recently developed powerful algorithms from the field of machine learning to determine which network properties are most predictive of neurological outcome. While there are innumerable examples of data-driven approaches across the graph theory literature, only a handful of studies have applied these algorithms to real-world networks. Recently this approach was used to discriminate between network-generating mechanisms, and then infer the correct mechanism that gave rise to several examples of gene regulatory and protein-protein interaction networks [14], [15]. This type of approach has not yet been applied to structural brain networks to predict the presence or absence of clinically significant developmental disruptions.

In this paper, we introduce the concept of using unsupervised and supervised learning algorithms for a data-driven approach to study brain connectivity networks and demonstrate their application in a cohort of neonates with clinically defined neonatal encephalopathy. The result is an algorithm that can accurately predict between good and poor neurological outcome, and which is robust over a broad range of parameters. We also identify network properties that are most discriminative between these two groups.

## Methods

### Study Patients and Imaging

The study was approved by the Committee on Human Research (CHR) of the University of California, San Francisco and was compliant with the Health Insurance Portability and Accountability ACT (HIPAA). Written and informed parental consent was obtained.

Diffusion tensor imaging (DTI) was performed on 24 six-month old infants who were born at gestational age >36 weeks and admitted to the UCSF Intensive Care Nursery with symptoms of neonatal encephalopathy. Although there are multiple complex associations and risk factors related to neonatal encephalopathy, our primary goal was to predict neurological outcome in patients with neonatal encephalopathy. Therefore, we matched our inclusion and exclusion criteria to previously established and well-defined criteria for this cohort. Specifically, the enrollment criteria were based on criteria used in the major clinical trials of hypothermia treatment for neonatal encephalopathy over the last decade [16]–[18]. The inclusion criteria were any one of the following: (i) umbilical cord arterial blood pH <7.1, (ii) umbilical cord arterial blood base excess >−10, (iii) Apgar score <5 at 5 minutes of age, (iv) post-asphyxia neonatal encephalopathy syndrome that includes stupor, diminished spontaneous movement, and hypotonia, and (v) seizures present on EEG from any acute symptomatic cause within 7 days of birth. Subjects were excluded if any of the following criteria were met: (i) syndromic diagnosis or congenital malformation (dysmorphic features, cardiac, genitourinary, or brain malformation based on physical exam or imaging) (ii) clinical, laboratory, or radiologic evidence of a congenital infection, (iii) inability to perform an MRI within 7 days of birth. Infants with congenital malformations or infections were excluded in order to avoid confounding factors for abnormal imaging or adverse neurodevelopmental outcome. Similar exclusion criteria were used in the major hypothermia clinical trials and in the oft-cited Sarnat and Sarnat article [19].

### Neurological Outcome

Our cohort is comprised of two separate classes: Neurologically Abnormal (NA) and Neurologically Normal (NN). Patients were defined as NA if they exhibited any of the following on subspecialty neurologic examination:

1. *Neuromotor score (NMS) >1*.
2. Seizures.
3. Abnormal neurological evaluation at 6 OR 12 months.

The NMS is a reproducible metric that demonstrates relatively high correlation with outcomes [6]. The score ranges on a scale from 0 to 5, with increasing probability of later poor outcome as NMS increases. As the sensitivity of this measure can be low, we also included (b) the presence or absence of seizure activity and (c) a 6-month and 12-month neurological evaluation. For the latter, a single board-certified pediatric neurologist examined the subjects based on predefined criteria [6] at 6 months and then again at 12 months, and then classified each subject as normal or abnormal at each age. If the findings of the evaluation were indeterminate, the neurologist designated the exam “unclear.” This final designation of “unclear” did not mean the patient had any abnormal neurological findings and is therefore not included in the “abnormal” or NA category.

Table S1 lists the scores for these measures for each of the 24 subjects. There were a total of 12 “NA” and 12 “NN” subjects.

### Data Acquisition

Subjects were scanned on a General Electric 3T EXCITE MR scanner using half-Fourier spin-echo (SE) echo planar imaging (EPI) diffusion tensor imaging (DTI) sequence with a field of view (FOV) of 24 cm ×4 cm, 72×128 matrix (half-Fourier with 8 overscans) reconstructed to 128×128 and zero-filled to 256×256, TE  = 57 ms, TR  = 9 s, 30 directions distributed by electrostatic repulsion, b-value  = 700 s/mm^2^ with a parallel imaging ASSET (Array Spatial Sensitivity Encoding Technique) acceleration factor of 2. Forty-five to fifty consecutive slices of 2 mm thickness were acquired though the entire brain. The scan time for DTI was approximately 4 minutes. The total time for each examination, which also included T_1_-weighted, T_2_-weighted, and spectroscopic imaging sequences, was approximately one hour. Subjects were scanned in an 8-channel adult head coil while under anesthesia, as is the standard of care at our institution.

### Assembling the Structural Connectome

We used a previously published computational pipeline for processing neonatal DTI data to construct structural networks [7]. Briefly, after acquisition of the diffusion-weighted images, the following steps were performed in sequence:

1. ***Quality assurance:*** Data affected by motion were rejected and remaining images were corrected for eddy current distortions and affine head motion. The FSL Brain Extraction Tool (BET) with a fractional intensity threshold of 0.5 was used to create a mask for subsequent tensor reconstruction [20].
2. ***Tractography:*** Diffusion tensor reconstruction and deterministic whole-brain streamline fiber tractography were performed using the Diffusion Toolkit [21] software package. The Fiber Assignment by Continuous Tracking (FACT) algorithm [22] was applied using the entire diffusion-weighted volume as the mask image with a threshold angle of 35°. Automated minimum and maximum thresholds were extracted from the histogram of the mask volume.
3. ***Surface extraction:*** Subcortical surface was extracted 2-4 mm below the cortex using the non-zero fractional anisotropy map and subsequent morphological operations (erosions and dilation) [23].
4. ***Parcellation:*** Automated, non-template based, parcellation of the cortical surface was performed using Recursive Zonal Equal Area Sphere Partitioning “equipartitioning” [24]. The number of nodes was chosen based on a network-driven automated method for determining the optimal number of nodes in six-month-old infants [25]. This method is detailed in the next section (see Methods: Non-template based parcellation).
5. ***Connectome construction:*** Computation of node-track and node-node connections was performed and the adjacency matrix constructed. The resulting graphs are undirected as diffusion MRI provides no information about directionality of the connections.
6. ***Binarization:*** The weights were binarized for the subsequent network analyses (see Methods: Network Embedding Space). We use a threshold of edge weight >0 for initial results but subsequently also vary the threshold to evaluate the robustness of the results.

### Non-template Based Parcellation

We have previously published a framework for assessing structural connectivity in the neonatal brain at any stage of development using a non-template based cortical parcellation [7]. An unbiased parcellation scheme is particularly relevant to the developing brain. Other commonly used methods are based on template-based registration with anatomic templates and landmarks derived from human cadaver or empirical studies on adult brains. Such templates may not be appropriate for the rapidly changing newborn brains, as they may introduce systematic biases. Although our framework does not require a template, it does require selection of the number of brain regions, *n*, into which the cortical surface is divided. These cortical brain regions will eventually be represented as nodes in a network. In our prior work we chose *n = 40*. As the decision for the optimal parcellation is nontrivial and can impact the values of derived network metrics [26], we developed a systematic data-driven approach to select *n*.

**Figure 1:**
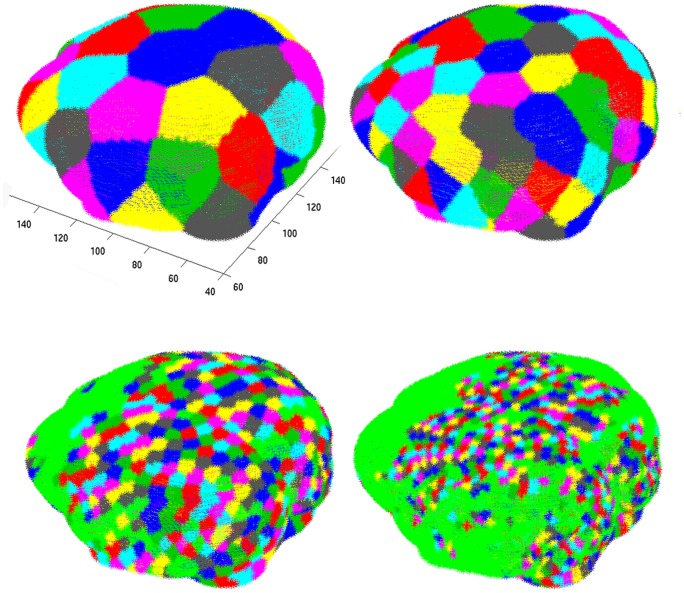
Brain parcellation for varying values of *n.* From left to right 40, 100, 1000, and 3000 nodes for a typical control subject. Colored brain regions represent nodes that are connected to the giant component. Green regions (which appear when *n>100*) represent nodes that are disconnected from the giant component.

In choosing a value for *n*, it is desirable to use the highest possible resolution parcellation (smallest cortical surface element) [27]. This is particularly true for the case of neonatal encephalopathy, where interregional connectivity may be modified in subtle ways. However, there is an inherent resolution limit to the acquired DTI data. As one increases *n*, the physical brain volume that each brain region represents becomes smaller. Eventually, a given brain region may become so small that the tracts connecting it to any other brain region are beyond the resolution of the imaging; in the reconstruction the corresponding node becomes disconnected from the rest of the network (see Figure 1). However, no brain region should be completely isolated from the rest of the network. These two opposing factors – requiring highest possible resolution parcellation while also requiring that every node be connected to the network – allowed us to define an unbiased value for *n* that is empirically derived from available data.

**Figure 2:**
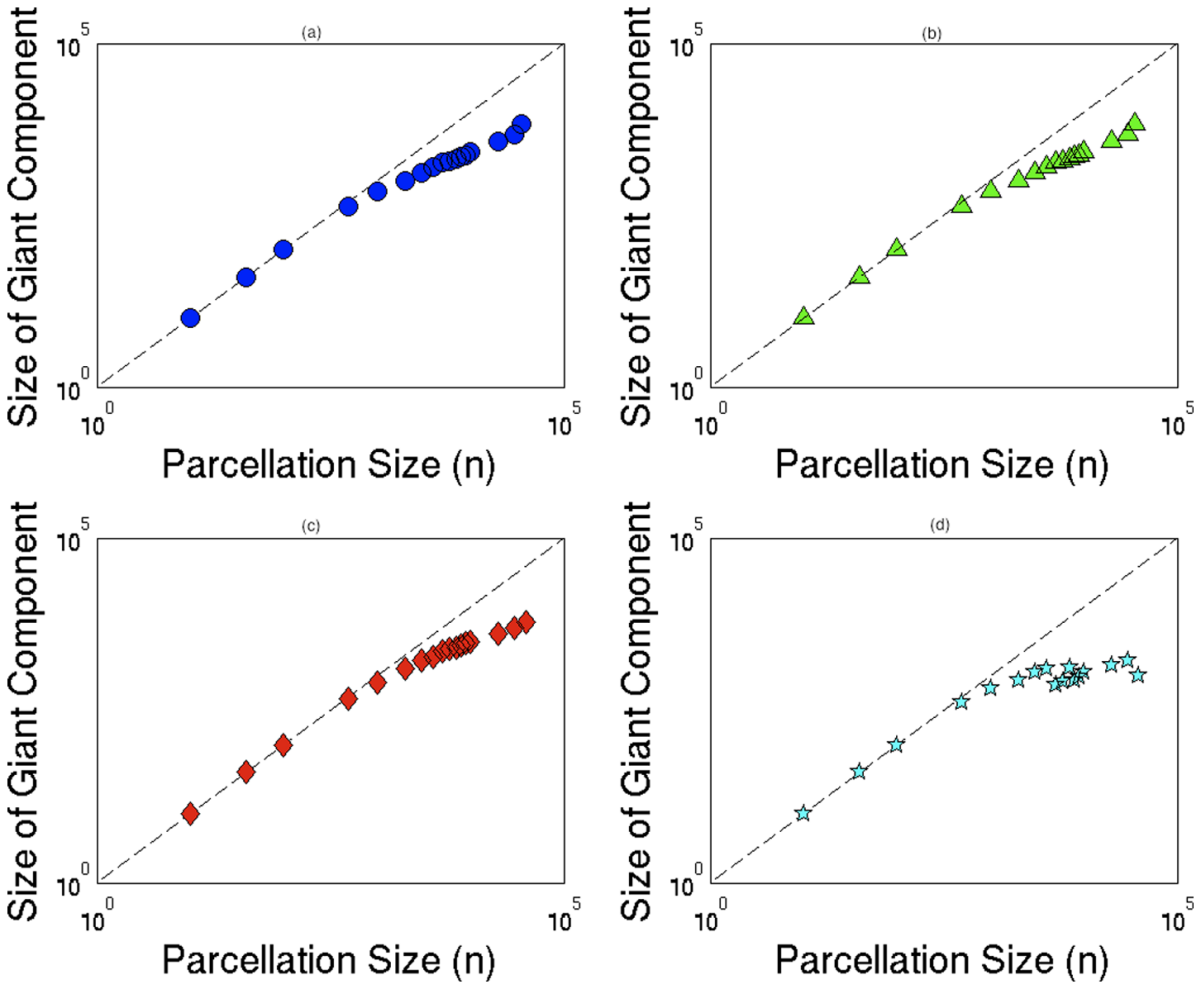
Giant component versus parecellation. Size of giant component *n_G_*, versus the size of parcellation, *n*, for four characteristic subjects. At *n>100*, the data points begin to fall off of the *n_G_ = n* line, motivating the use of *n = 100* as a parcellation size which maximizes resolution while preserving brain connectivity.

For each subject, we made multiple constructions of the connectome by increasing the value of *n*. We sought the maximum *n* for which the resulting network maintains connectivity over the entire brain. This approach is demonstrated on one subject with normal neurological outcome in Figure 1. For parcellations where *n>100*, we observed isolated brain regions. We performed the same set of operations on each of the 24 subjects. We defined *n_G_*, the number of nodes in the largest connected component (also known as the giant component) [28]. Because all of the brain regions were expected to be connected to the giant component, we imposed the constraint that *n_G_ = n*, and picked the largest value of *n* that satisfied this requirement. Four typical examples of the relationship between *n_G_* and *n* are illustrated in Figures 2a–d. Note that for all subjects at *n>100*, the networks began to fall off of the *n_G_ = n* line. We thus set *n = 100* for all 24 subjects, maximizing resolution while preserving total brain connectivity. The determinates of the ultimate value of *n* derived using this approach are complex, and depend upon the parameters used for diffusion acquisition (including b value and spatial resolution), the tractography method, and the underlying scale at which connectivity is defined in the subjects of interest.

### Network Properties

The brain connectivity network that we constructed consists of a set of *n* brain regions (vertices) that are connected by tracts (edges). The network is conveniently represented as an adjacency matrix *A*, where *A_ij_ = e_ij_* (the edge (tract) connecting region *i* with region *j*) and *e_ij_ = 0* if there exists no edge between regions *i* and *j*, and *e_ij_ = 1* otherwise (see Table S2 for summary of variables used in the manuscript).

We define the degree of a brain region *i* as *d_i_ = Σ_j_A_ij_*, which for our unweighted networks is equivalent to the number of tracts incident on region *i*. Let *P(d)* be the fraction of regions in the network with degree *d*. Additionally, we defined the following graph theoretic properties as they have been widely used in the literature:

Clustering coefficient [29]:
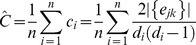
(1)where 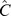 is the average clustering coefficient over all *n* regions, *c_i_* is the local clustering coefficient for the *i^th^* region, *d_i_* is the degree of the *i^th^* region, and *e_jk_ = 1* if *j* and *k* are neighbors of the *i^th^* region and are connected to each other (that is, *i,j,k* form a triangle).

Mean geodesic length [30]:
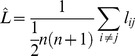
(2)where 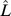 is the geodesic length averaged over all *n* regions, and *l_ij_* is the geodesic distance from region *i* to region *j*.

Transitivity [30]:
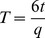
(3)where *t* equals the number of triangles in the network and *q* equals the number of paths (consecutive tracts) of length two.

### Network Embedding Space

Systematic enumeration of subgraphs has been well-studied in the sociology literature [31] and has more recently been applied to gene regulatory networks [9]. Typically these approaches assume a null model and test for statistical significance. Alternatively, the raw subgraph counts can be used as a network embedding space, in which a given network is mapped to a high-dimensional space. It is this network embedding space which can then be used as the input space for a machine learning algorithm to discriminate between two classes of networks. This approach was previously used successfully for analyzing the network-generating mechanisms of protein-protein interaction networks [14], [15].

There are multiple ways to define a cut-off in subgraph size, including number of edges (in this model, tracts), number of vertices (in this model, regions), and number of edges in a walk, where a walk is defined as a sequence of tracts, such that each adjacent pair of tracts in the sequence share at least one region. A walk of length *x* therefore means any such sequence of *x* tracts. Similar to prior work [15], we count all possible subgraphs that can be constructed by a walk of length eight, which yields a total of 149 non-isomorphic subgraphs. We note that the mean geodesic length for most of the networks ranged from 3 to 4 and the mean degree ranged from 4 to 8. The subgraphs generated from walks of length eight can therefore span large parts of the network.

The algorithm proceeds by counting all possible walks in the network of length eight and then grouping isomorphic subgraphs. We use freely available source code at http://sourceforge.net/projects/stat-mod-net.

### Unsupervised Learning Algorithm

A standard unsupervised learning tool for dimensionality reduction is *principal components analysis*, or **PCA** [32]. PCA is a simple, non-parametric method to identify the subspace in which the data approximately lies.

Each subject's 100-region network is mapped to a 149-dimensional space of subgraph counts so that our initial dataset of 24 100-region networks is now represented as a data matrix *X* (*NxM*) where N is the number of subjects and M is the number of dimensions (see Table S2 for a summary of variables used in the manuscript).

Briefly, we zero mean our data and then construct the covariance matrix. Next we compute the matrix of eigenvectors that diagonalize the covariance matrix and sort the columns of the eigenvector matrix in order of decreasing value of the corresponding eigenvalues.

Finally, we select a subset of the first *p* eigenvectors as basis vectors and project the data onto the new basis
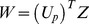
(4)where *U_p_* represents the first *p* columns of *U* and *Z* is the data matrix with zero mean.

### Supervised Learning Algorithm

A standard supervised learning algorithm which has been used with great success across a number of disciplines is the *support vector machine*, or **SVM** [33]. This technique empirically separates two classes from each other in a high-dimensional feature space. We use a freely available Matlab-implementation of SVM (http://people.kyb.tuebingen.mpg.de/spider/main.html)– an object-oriented interface that runs the C-implemented LIBSVM library [34]. This package uses a working set selection method to solve the convex programming problem with Lagrangian, *L*,
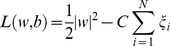
(5)and
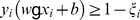
(6)where f = w•x+b is the equation of the separating hyperplane, xi are the training examples, yi ∈ {−1,1} are the class labels, and ξi are positive slack variables, µ i = 1,…,N. For non-separable (overlapping) data, ξi allow some of the data to be misclassified, while the tuning parameter, C, controls how many outliers to allow and determines the trade-off between small errors and large margin (see Table S2 for summary of variables used in the manuscript).

We perform nested cross-validation [35] for model selection to choose the linear SVM parameter *C*, using leave-one-out cross-validation for the inner loop and a two-fold cross-validation to estimate the generalization error in the outer loop.

To approximate statistical significance of our classification error statistic, we performed non-parametric permutation testing [36]–[38]. Specifically, we randomly permuted the class labels (a total of 1000 times) and then performed the nested cross-validation on each new permuted dataset, thus generating a distribution of test errors on random data. Constructing an empirical cumulative distribution over the test errors allows us to compute a p-value for the reported test error (Figure S1).

### Discriminative Feature Extraction

After having trained a successful SVM, which demonstrates good testing error, it is possible to extract the most discriminative features from the original input. This problem has also been explored in depth in the machine learning literature. One approach, which we employ here, is to use the SVM on the features for Recursive Feature Elimination (RFE), an algorithm that was first applied to a gene expression dataset [39]. Briefly, the algorithm iteratively trains the classifier, computes the change in the cost function (typically the mean-squared error) caused by removing a given feature, and removes the feature that gives the smallest change. The freely available Matlab-implementation of SVM (http://people.kyb.tuebingen.mpg.de/spider/main.html) includes a feature extraction tool to implement this algorithm.

An alternative technique is first to select principal components (PCs), and then to use the chosen PCs to select a feature subset. Multiple approaches have been proposed to select a feature subset using the PCs [40], [41]. The so-called “B4” method is an intuitive and computationally feasible technique which has demonstrated consistent success in the literature [42]. This method involves associating one subgraph with each PC by choosing the subgraph that contributes the most to the PC (has the highest coefficient in absolute value). Selecting PCs can be accomplished either by using a feature extraction algorithm such as the RFE algorithm described above, or by directly selecting the PCs with the highest eigenvalue. In the latter, however, there is no guarantee that the selected PCs will identify discriminative features.

We summarize the three approaches we have described here:

1. RFE-raw: perform SVM-based recursive feature elimination on the raw subgraph features.
2. RFE-pca: perform SVM-based recursive feature elimination on the PCs and associate each selected PC with a subgraph using the “B4” method.
3. topPCA: select top PCs (PCs with corresponding highest eigenvalues) and associate each selected PC with a subgraph using the “B4” method.

## Results

### Network Properties

Initially we constructed 100-node connected networks for each of the 24 subjects and measured average degree distribution, clustering coefficient, mean geodesic length, and transitivity. Means and standard deviations for the four measures are given in Table 1 for the two groups, neurologically normal (NN) and neurologically abnormal (NA). No significant differences are seen between the two groups in average degree (5.7±1.1 compared with 5.6±1.0), mean geodesic length (3.4±0.3, compared with 3.5±0.5), average clustering coefficient (0.34±0.04, compared with 0.33±0.04), and transitivity (0.33±0.03, compared with 0.32±0.01). Values are consistent with recently published reports of network metrics on pediatric brains [7], [43], [44].

**Table:** Table 1. Typical network metrics.

	NORMAL	ABNORMAL
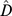 **Average Degree,**	5.7±1.1	5.6±1.0
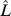 **Mean Geodesic,**	3.4±0.3	3.5±0.5
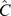 **Clustering Coefficient,**	0.34±0.04	0.33±0.04
**Transitivity, ** ***T***	0.33±0.03	0.32±0.01

Averaged over the 12 subjects in each group (normal and abnormal). The two groups are indistinguishable in their average degree (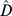), mean path length (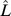), average clustering coefficient (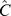), and transitivity (*T*).

**Figure 3:**
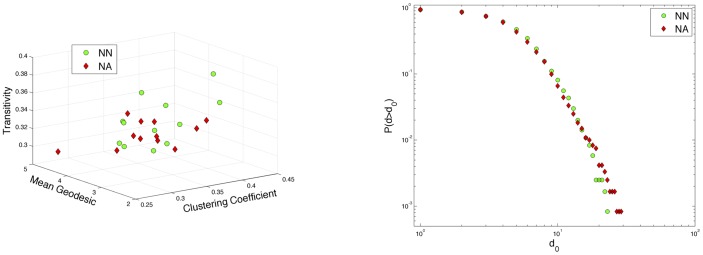
Non-separability in typical network measures. Neurologically normal (NN) and neurologically abnormal (NA) networks (a) inseparable in the three-dimensional space defined by the transitivity, clustering coefficient, and mean geodesic and (b) exhibiting complete overlap in the cumulative distribution function *P(d>d_0_)*.

In Figure 3a we map the two groups of networks to a three-dimensional space defined by the clustering coefficient, mean geodesic length, and transitivity. Similar to our prior work, we do observe a general trend where increased clustering coefficient, decreased mean path length, and increased transitivity are correlated with normal neurological outcome. However, it is impossible to define a hyperplane that can reliably distinguish between normal and abnormal neurological outcome. The two groups also exhibit marked overlap in their degree distribution (demonstrated by the cumulative density function in Figure 3b).

### Unsupervised Learning

**Figure 4:**
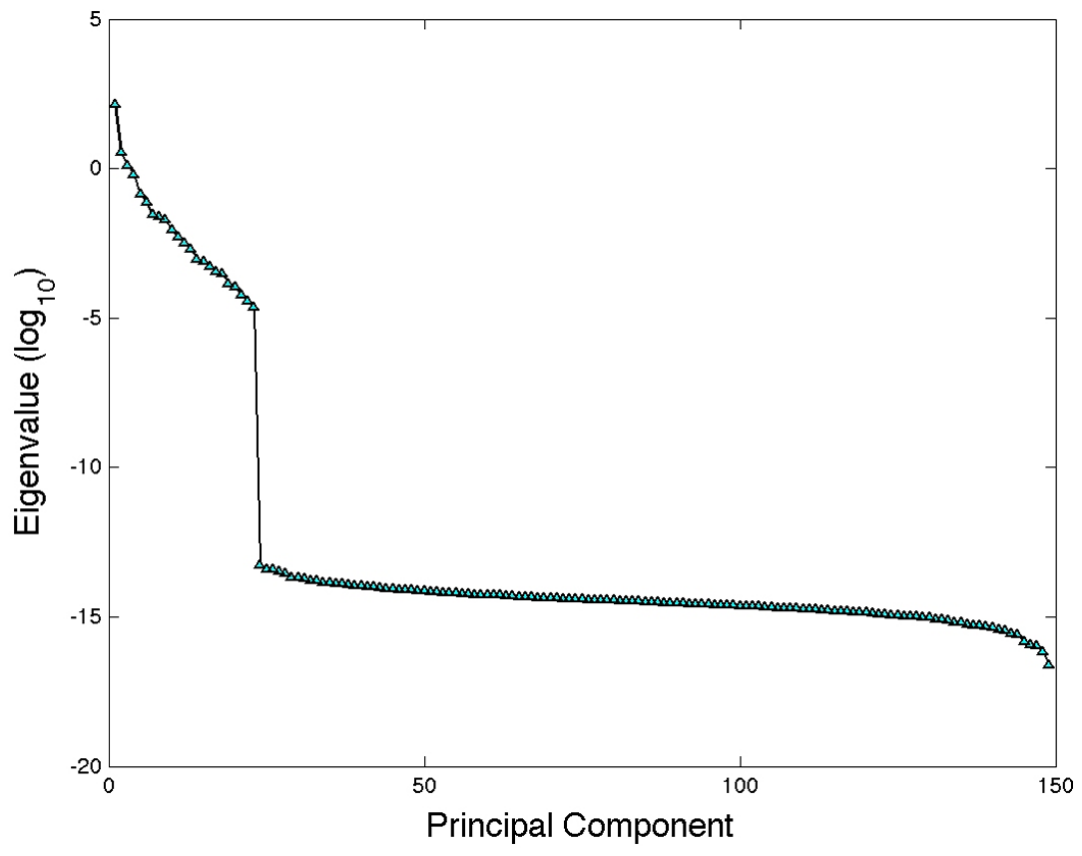
Eigenvalues of the principal components. Eigenvalues of the principal components demonstrate an abrupt drop near the 20^th^ principal component, suggesting a natural cutoff for dimensionality reduction.

Rather than pick network properties of interest as the basis for which to distinguish between clinical phenotypes, we can systematically catalog many network metrics automatically and use dimensionality reduction to identify discriminating features. For this approach, we enumerated all subgraphs that can be constructed by a walk of up to eight steps (the networks have average path length of 3.5 so that subgraphs included in this space can traverse large parts of the networks). We then performed PCA on the resulting 24×149 data matrix, *X*, by subtracting the mean along each dimension, computing the covariance matrix and then performing the eigendecomposition of the covariance matrix. The eigenvalues of the principal components are plotted in Figure 4 and exhibit a natural cutoff near 20 for dimensionality reduction.

**Figure 5:**
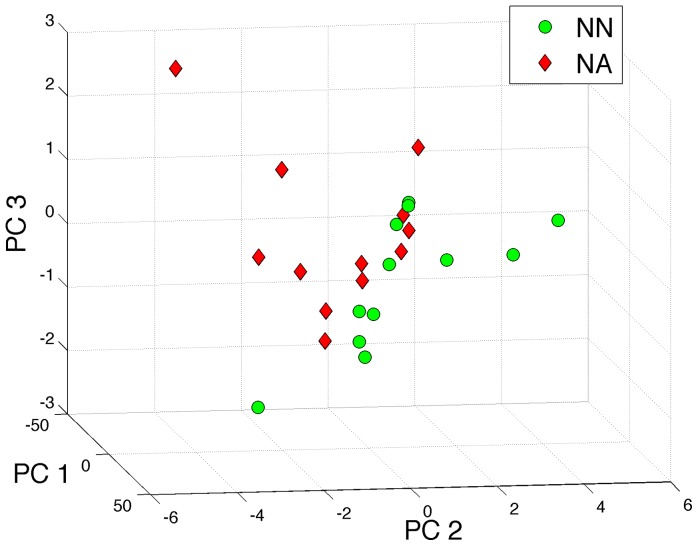
First three principal components. The first three principal components composing a three-dimensional shadow of the 149-dimensional space with a notable trend separating the two groups neurologically normal (NN) and neurologically abnormal (NA) has emerged from the PCA.

To visualize the data in the resulting subspace, we plot the two groups in the three dimensional shadow defined by the first three principal components (Figure 5). A striking trend is revealed, suggesting the two classes may indeed be separable in this embedding space, despite their complete overlap in the four network properties described above. Notably, we have not introduced any information about the groups thus far. The trend shown in Figure 5 has naturally emerged from the data and shows that differences between the two groups can explain a large portion of the variation in the data.

### Supervised Learning

**Figure 6:**
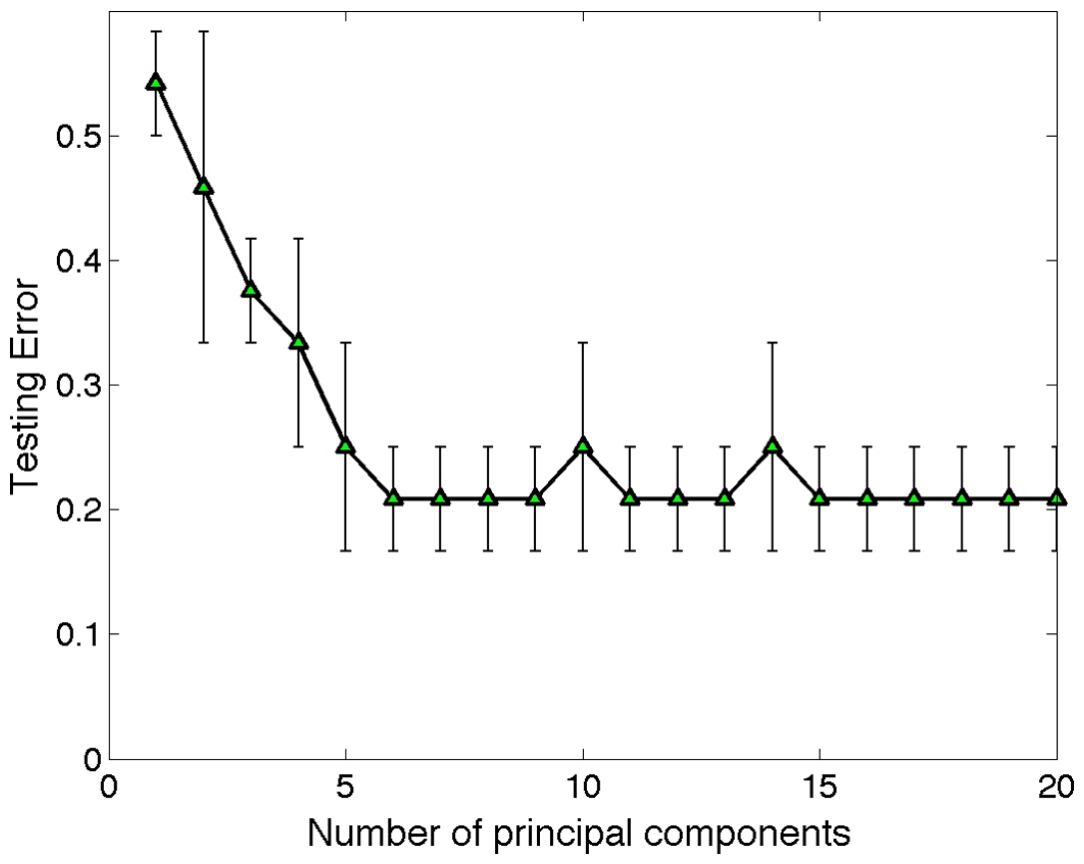
Generalization error vs number of principal components. Estimate of the generalization versus the number of principal components employed in the SVM. Testing error is obtained by using a nested cross-validation scheme in which the inner loop is used to select the SVM parameter, *C*, and the outer loop is used to estimate the generalization error.

Motivated by the apparent linear separability suggested by the PCA analysis above, we next *quantified* how reliably separable the two classes are. We trained a linear SVM and used a nested cross-validation scheme. In the inner loop, we performed cross-validation to choose the *C* parameter that gave the best testing error. However, this testing error had an inherent bias [35], which we avoided by employing an outer loop of cross-validation to estimate the true generalization error–counting the number of misclassifications on a hold-out set from each inner loop and averaging over the folds. Testing accuracy as a function of the number of principal components is shown in Figure 6. We were able to predict the presence of neurological abnormality with 0.79+/−0.04 testing accuracy (*p-value = 0.002*), where we determine statistical significance using non-parametric random permutation testing (Figure S1). Performing the classification using the four typical network properties yielded a test error of 0.51+/−0.07. Combining the four typical network properties with our subgraph space yielded a test error of 0.33+/0.08, which was slightly worse than the classification error using just the subgraph space.

### Dependence on Binarization Threshold

We tested the stability of the classifier over a 50-fold range of threshold values (Figure S2a). As a comparison, we also tested the stability of the typical network properties over the same range of threshold values (Figure S2b). As the networks are disconnected at higher threshold levels, the mean geodesic length is computed on the giant component [30]. We note this classification result is stable with statistical significance (p-value<0.05) over nearly a 10-fold range of threshold values (Figure S2a). At higher threshold levels the networks become increasingly sparse and disconnected resulting in changes in all four of the typical network properties (Figure S2b).

### Feature Selection

**Figure 7:**
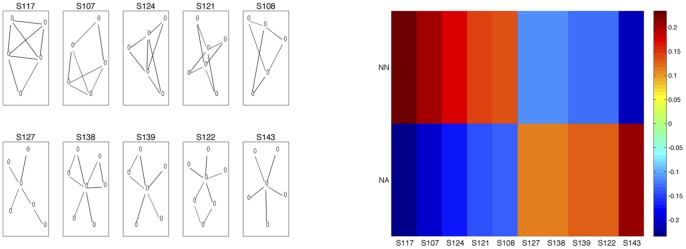
Recursive feature elimination. (**a**) Top 10 subgraphs from the 149-dimensional feature space where ranking is based on recursive feature elimination (using 10-fold cross-validation, C = 50, and pruning to 10 features). (b) Standard scores for top 10 subgraphs in (a), averaged over the 12 subjects for each of the two groups, neurologically normal (NN) and neurologically abnormal (NA).

While our approach emphasizes the classification task of discriminating pathology, we can also glean some information regarding the network properties that are the most discriminating. The PCA demonstrated qualitatively that the difference between the two groups was largely described by the variation in the data. However the PCs represent linear combinations of the network properties and so may be difficult to interpret. Often multiple properties contribute to each PC. One strategy (RFE-raw) for subset feature selection involves using the SVMs to discover discriminative features [45]. We applied RFE-raw to our classification problem to obtain a rank order for the 149 features. The top 10 subgraphs from this ranking are depicted in Figure 7a. Figure 7b shows the standard scores for each of these 10 top subgraphs averaged over the two groups, NN and NA. A review of these top ten topologies demonstrated a consistent trend where the NA group had relative depletion in the number of subgraphs with overlapping 3-node or 4-node cycles compared with the NN group (subgraphs S124, S108, S121, S117, and S107). Instead, the NA group had subgraphs with hubs of degree 4–6 connecting to branching chains or cycles; but importantly the cycles did not overlap (subgraphs S138, S143, S122, S139, and S127). Classifying the two groups using just these top 10 subgraphs gave a classification error of 0.23+0.08 (p = 0.005).

We also used the RFE-pca and topPCA feature selection strategies to choose the top 10 subgraphs. Classifying the two groups using the 10 subgraphs from each method gave classification errors of 0.33+/−0.10 (p = 0.05) and 0.31+/−0.11 (p = 0.05), respectively. Results are summarized in Table 2. Figures S3 and S4 also show the top ten subgraphs selected by the RFE-pca and topPCA methods. Overlap between the two PC–based subsets and the rfe-raw subset again highlights the observation that the variance in the data is to a large extent explained by the two classes.

**Table:** Table 2. Generalization Errors.

	Test Error	p-value
**RFE-raw**	0.23+/−0.08	0.005
**RFE-pca**	0.33+/−0.10	0.05
**topPCA**	0.31+/−0.11	0.05

Summary of classification results and p-values for the three subset feature methods (10 subgraphs).

## Discussion

We have presented a data-driven approach to predicting neonatal encephalopathy based on structural networks derived from DTI data and demonstrate low testing error (21%+/−4%). The results illustrate the robustness of this approach: despite noisy data [46] and template-free parcellation, our generalization error was statistically significant over a nearly 10-fold range of threshold values.

Several recent studies in children have reported correlations between network properties of structural connectomes and clinical parameters [43], [44], [47], [48], in addition to correlation between network integration and age in structural connectomes of children at various ages [25]. Wu et al report correlations between network properties and age, gender, and IQ in functional brain networks of normal children [49]. Our approach differs from others in two important ways. First, rather than looking for correlations with clinical measures, our approach exploited machine learning algorithms to *predict* clinical outcome. Second, rather than selecting pre-determined network properties of interest, our approach systematically enumerated many network properties and used our classifier results to *learn* which network properties were most discriminative and therefore clinically relevant. In this way, more complex associations were discovered – for example, the finding that neonates who are clinically neurologically abnormal have fewer subgraphs with overlapping cycles. To date, we are not aware of any such *data-driven* approaches in the human connectome literature.

### Study Design

Overfitting is a major issue when the number of observations is small relative to the number of parameters, such as our dataset containing a relatively small sample size compared to the high dimensional subgraph space. Cross-validation mitigates this by separating the data into training and testing sets and reporting only the prediction accuracy on the testing set. As cross-validation provides an estimate of the true prediction accuracy, we also reported the estimated standard error of the reported prediction accuracy [32]. Another limitation is the heterogeneity of our study cohort –the subjects share the clinical diagnosis of neonatal encephalopathy, but multiple potential etiologies exist for this disorder. This problem is ubiquitous in the literature [50] studying this population. Finally, we are limited by the cross-sectional nature of the study. Whether our findings of differences between the NN and NA connectomes holds true for older children, or these differences can be detected at earlier ages, will be determined by future studies.

### Parcellation

Reconstructing structural brain networks of neonates from DTI data is an active area of research, and template-free parcellation is of particular interest since current atlases are derived from cadaver studies on adult brains. Moreover, the neonatal period is a very dynamic phase of neurodevelopment [48]. Building on our recent work on template-free parcellation [7], we have introduced a graph-theoretic technique for choosing the “optimal” (maximizing resolution while preserving brain connectivity) number of brain regions. It is well-established that the choice of resolution (the number of brain regions) can dramatically affect network properties [26]. We were able to avoid this issue, as our resolution arises from the data. However, our technique of starting with an equipartitioning of the cortex assumes a certain shape for the brain regions. The dependence of the network topology on this shape is uncertain and requires further investigation. Interestingly, the number of brain regions in this approach depends on the imaging parameters, and in this sense represents a measure of the quality of the imaging – the finer the parcellation achieved, the better the quality.

### Threshold

We have used a threshold for the edges (edge weight>0) for the construction of the connectome. The low threshold may result in noisier data since low weight edges may represent “false” tracks that are then considered equivalent to “real” high weight tracks. The classification task is therefore made more difficult as the networks would all appear more similar to Erdos-Renyi networks [15]. On the other hand, a high threshold could also make the classification task more difficult as the networks become increasingly sparse and disconnected. We demonstrated stability of network properties as well as our classification over a 10-fold range of threshold values. However, the relationship between the threshold, the parcellation, and ultimately the classification, is complex, as for a given parcellation, it is also possible to define an “optimal” threshold that will allow coverage of the entire brain with no disconnected components. Therefore the choice of threshold remains an active area of research in the community at this time.

### Subgraph Space

Subgraph census has its origins in the sociology literature [31] and has more recently been adapted to study various real-world networks [9], in which the subgraph counts are compared with a presumed null model (typically the configuration model [30]) to identify statistically significant subgraphs, dubbed “network motifs” [51]. This technique has also been applied to structural human connectomes [52]. In a recent review article, Kaiser discusses some interesting limitations of applying network motif analysis in the human connectome including the fact that such networks are undirected and a more subtle issue related to densely connected modules [53]. It should be emphasized that the approach we have presented here does not presume a null model and does not involve identifying statistically significant networks. We use the subgraph census as a convenient embedding space for the networks, allowing discrimination between networks from two clinically distinct groups.

### Network Classification

This work is inspired by a similar approach first introduced in the molecular network community to infer the network-generating mechanism that gave rise to protein-protein interaction network [14], [15]. We have applied the same *explicit* network embedding space to brain connectomes. In the machine learning literature there has been much interest in exploiting the power of kernel methods [54] for structured data, including networks. A kernel is a measure of similarity between two samples and the “kernel trick” allows one to *implicitly* map the data into a high-dimensional feature space. A general framework for kernels between graphs [55], [56] has recently been presented and adapting such an approach to brain networks may be an interesting direction to pursue. Alternatively, an explicit network embedding space which also can accommodate weighted networks and is based on linear combinations of walks on a network has been proposed and applied to gene regulatory network analysis [57]. Both of these approaches may be adapted to future studies investigating brain network pathology.

### Feature Extraction

Feature selection revealed an interesting trend–abnormal subjects tend to have lower standard scores of subgraphs with overlapping 3-node or 4-node cycles, and instead have higher standard scores of subgraphs with hubs whose neighbors are poorly connected. This would appear to suggest that measures such as clustering coefficient and transitivity would be different in the two groups. Indeed, in our previous work we have demonstrated that the clustering coefficient is anti-correlated with NMS [7]. However, these measures do not reliably separate the two classes. Instead, we find that the most discriminative features are more complex structures that include overlapping cycles. Interestingly, previous work has also observed overlapping cycles as a recurring motif in other naturally occurring networks [51], [57].

### Conclusion

We have presented a method based on diffusion tractography data to predict neurological outcome in subjects at high risk for developing neurological deficit. Our approach is data-driven and our results are statistically significant and robust. Finally, it has not escaped our attention that this technique may be well suited to study a broad range of brain pathologies which are conjectured to affect connectivity – including neurodegenerative, neurodevelopmental, and psychiatric diseases.

## Supporting Information

Figure S1 **Statistical Significance.** Histogram of test errors derived from SVM nested cross-validation on 1000 instances of the dataset with randomly permuted class labels. Red star indicates test error on the true dataset (21%), which corresponds to a p-value of 0.002.
(TIFF)

Figure S2 **Threshold dependence.** (**a**) Edge weight binarization was performed over an approximately 50-fold range of threshold values. Generalization error remained stable and statistically significant over nearly a 10-fold range of threshold values. (**b**) The four typical network properties also show similar stability over a 10-fold range of threshold values. At higher threshold values the networks become more sparse and demonstrate increasing geodesic length and decreasing clustering coefficient. (Geodesic length is defined here on the giant component [30] of each network since the networks become disconnected at higher threshold levels).
(TIFF)

Figure S3 **Recursive feature elimination (PCA).** (**a**) Top 10 subgraphs from the 149-dimensional feature space where ranking is based on recursive feature elimination in PCA space and then mapping top 10 selected PCs to most representative subgraphs. Classification accuracy for this 10-subgraph space was 0.33+/−0.07 (p = 0.05). (**b**) Standard scores for top 10 subgraphs in (a), averaged over the 12 subjects for each of the two groups, neurologically normal (NN) and neurologically abnormal (NA).
(TIFF)

Figure S4 **Recursive feature elimination (PCA).** (**a**) Top 10 subgraphs from the 149-dimensional feature space where ranking is based on top 10 PCs and then mapping these to most representative subgraphs. Classification accuracy for this 10-subgraph space was 0.31+/−0.10 (p = 0.05). (**b**) Standard scores for top 10 subgraphs in (a), averaged over the 12 subjects for each of the two groups, neurologically normal (NN) and neurologically abnormal (NA).
(TIFF)

Table S1 **Clinical measures of neurological outcome**. We define classes “−1” (abnormal neurological outcome or NA) and “1” (normal neurological outcome or NN). Class “1” is defined as having one or more of any of the following: NMS>1, seizures, or abnormal neurological evaluation (NE) by a pediatric neurologist at 6 months or 12 months. Missing evaluations at 12 months had not yet been completed.
(DOCX)

Table S2 **Summary of variables used.**
(DOCX)
