# Supplementary figures and images for: A Machine Learning Approach to Automated Structural Network Analysis: Application to Neonatal Encephalopathy
Source: PLoS One. 2013 Nov 25;8(11):e78824. doi: 10.1371/journal.pone.0078824 (PMC3840059; doi:10.1371/journal.pone.0078824)

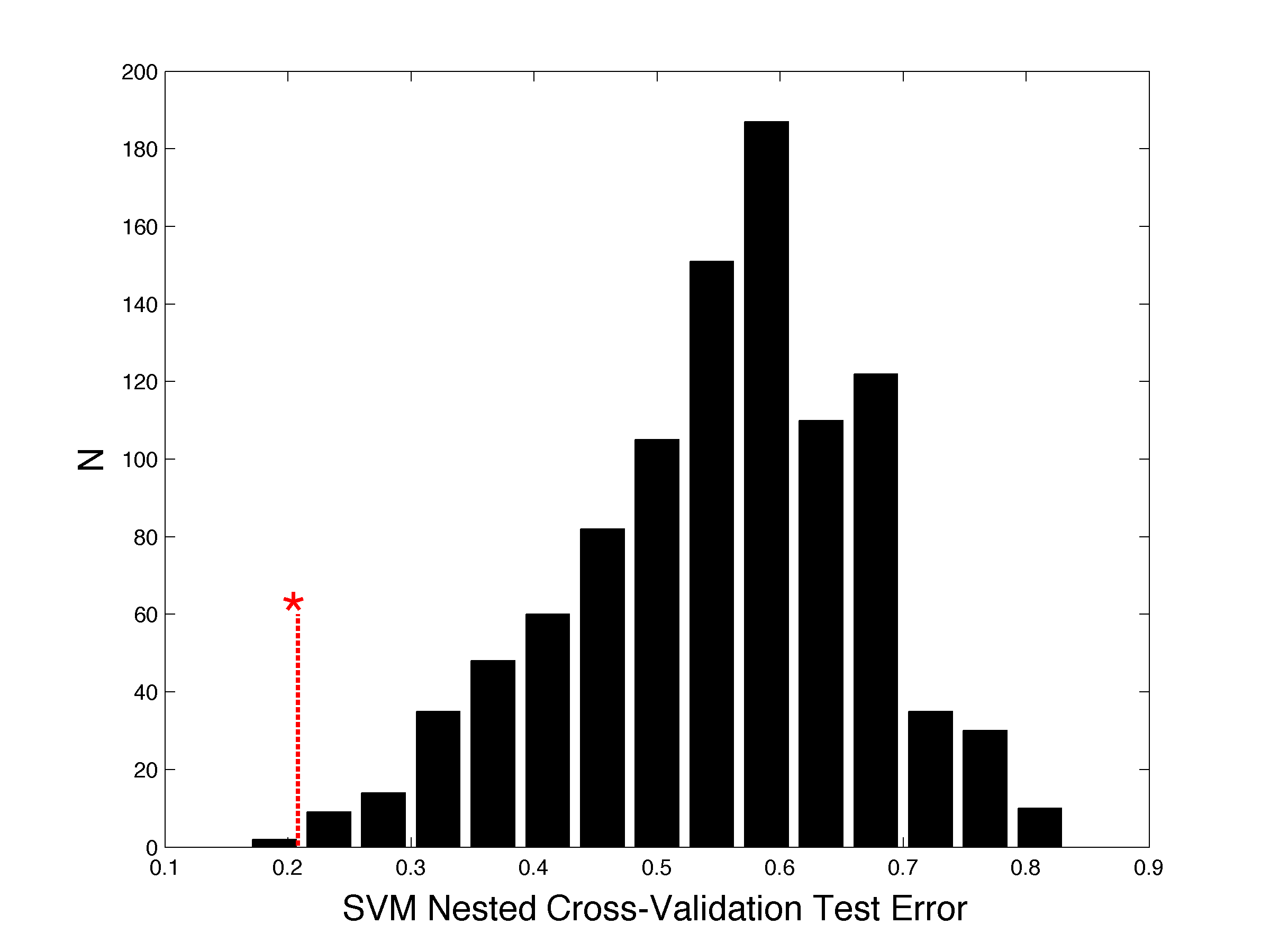

Supplement: Figure S1 — Statistical Significance. Histogram of test errors derived from SVM nested cross-validation on 1000 instances of the dataset with randomly permuted class labels. Red star indicates test error on the true dataset (21%), which corresponds to a p-value of 0.002. (TIFF) [file pone.0078824.s001.tfif]

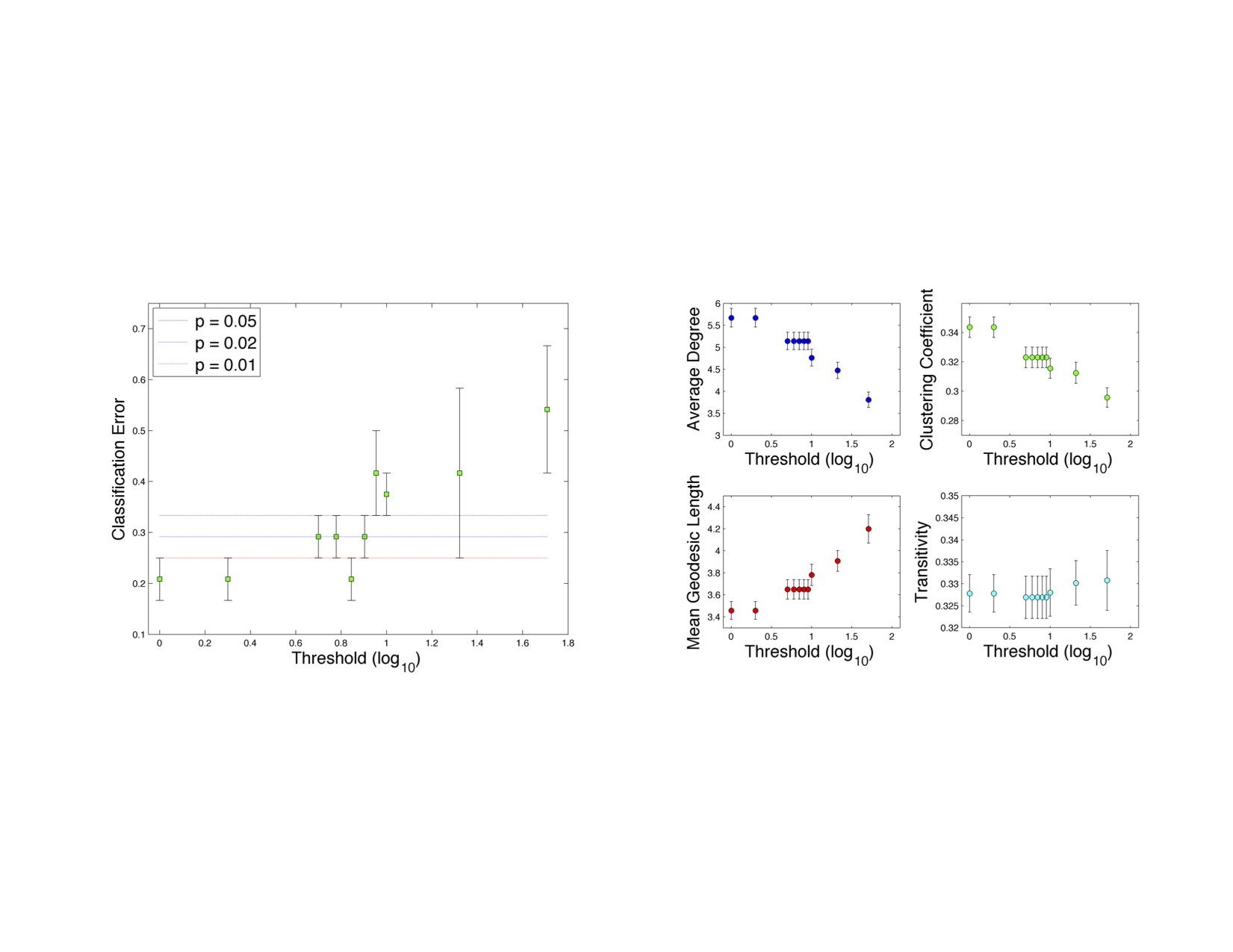

Supplement: Figure S2 — Threshold dependence. (a) Edge weight binarization was performed over an approximately 50-fold range of threshold values. Generalization error remained stable and statistically significant over nearly a 10-fold range of threshold values. (b) The four typical network properties also show similar stability over a 10-fold range of threshold values. At higher threshold values the networks become more sparse and demonstrate increasing geodesic length and decreasing clustering coefficient. (Geodesic length is defined here on the giant component [30] of each network since the networks become disconnected at higher threshold levels). (TIFF) [file pone.0078824.s002.tiff]

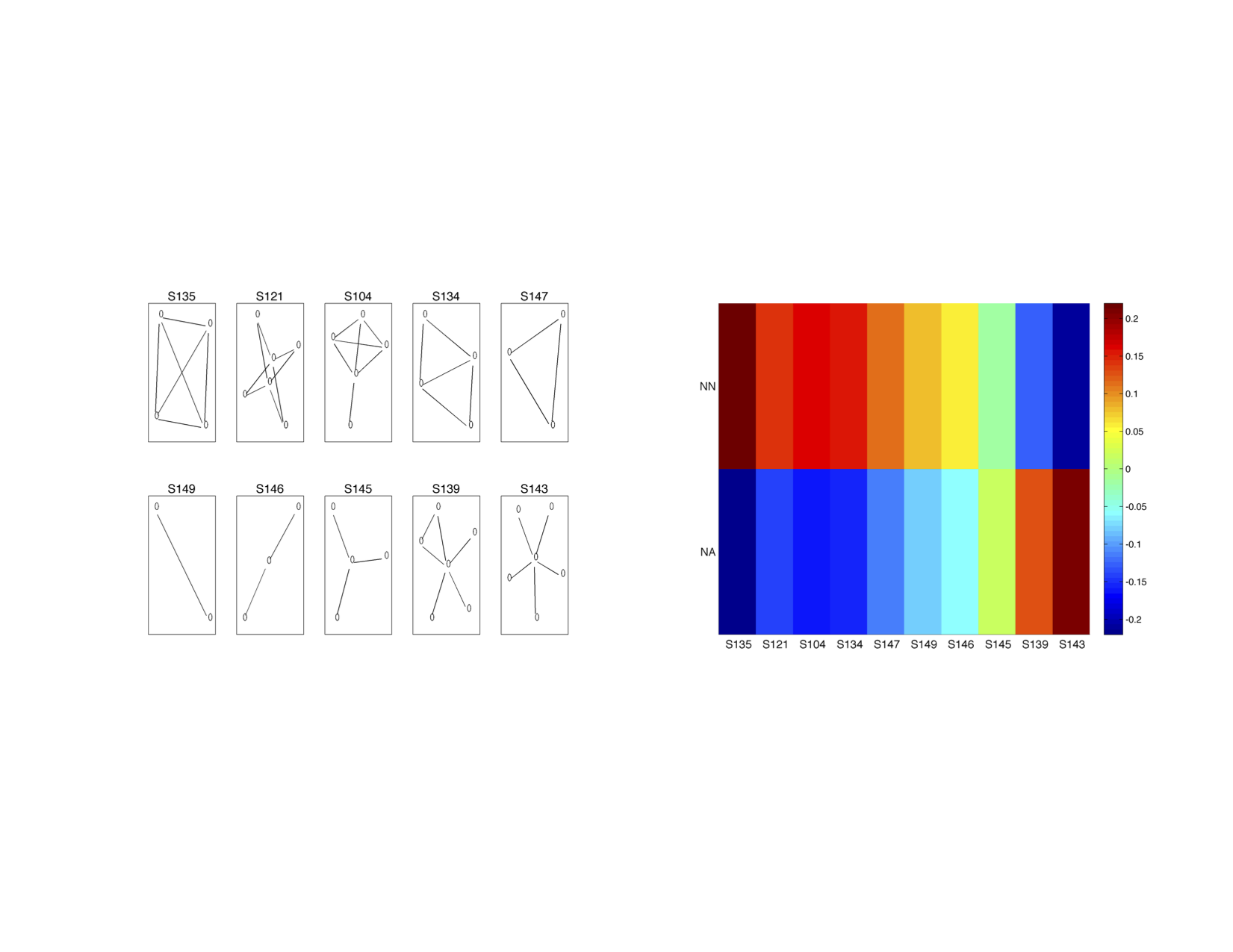

Supplement: Figure S3 — Recursive feature elimination (PCA). (a) Top 10 subgraphs from the 149-dimensional feature space where ranking is based on recursive feature elimination in PCA space and then mapping top 10 selected PCs to most representative subgraphs. Classification accuracy for this 10-subgraph space was 0.33+/−0.07 (p = 0.05). (b) Standard scores for top 10 subgraphs in (a), averaged over the 12 subjects for each of the two groups, neurologically normal (NN) and neurologically abnormal (NA). (TIFF) [file pone.0078824.s003.tiff]

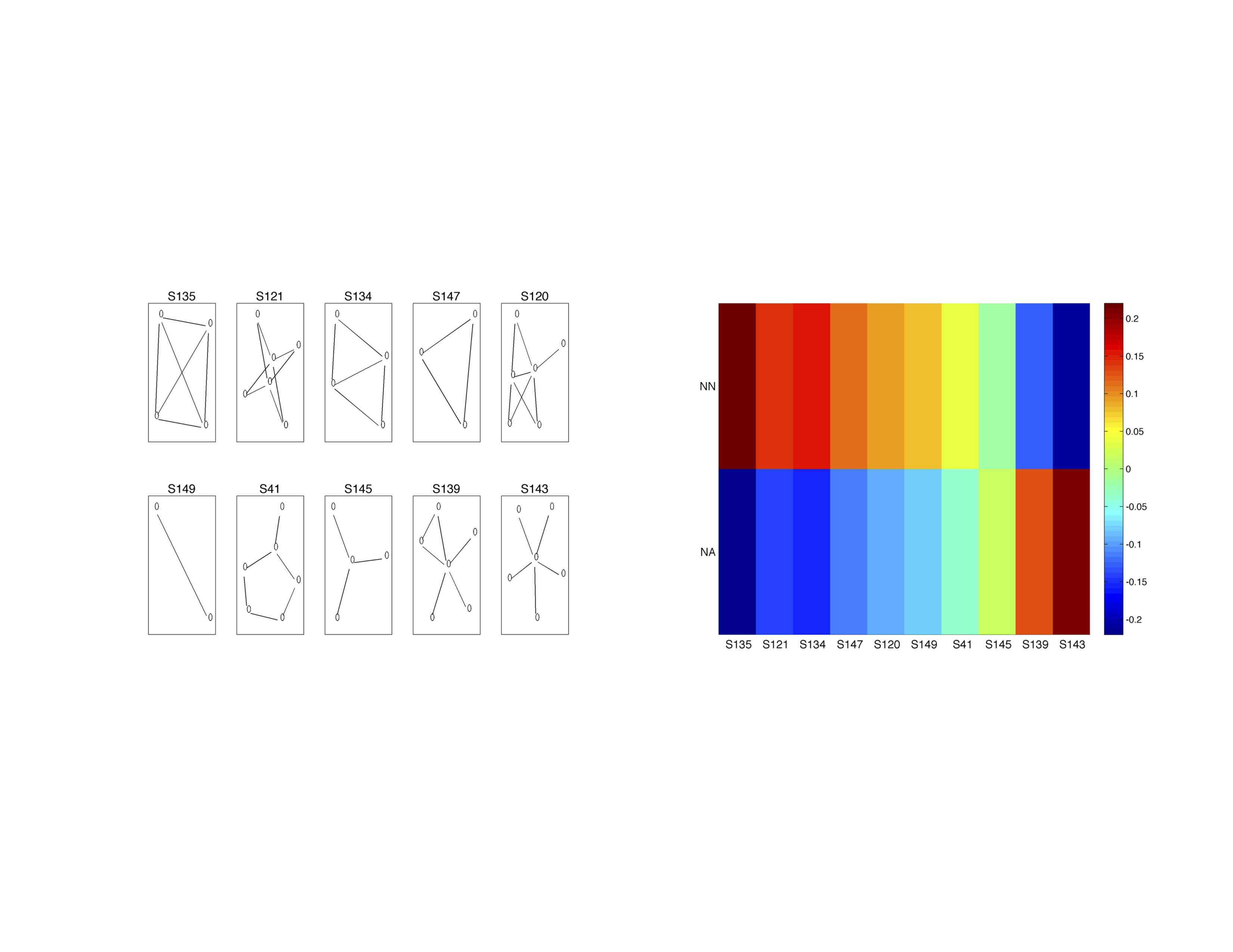

Supplement: Figure S4 — Recursive feature elimination (PCA). (a) Top 10 subgraphs from the 149-dimensional feature space where ranking is based on top 10 PCs and then mapping these to most representative subgraphs. Classification accuracy for this 10-subgraph space was 0.31+/−0.10 (p = 0.05). (b) Standard scores for top 10 subgraphs in (a), averaged over the 12 subjects for each of the two groups, neurologically normal (NN) and neurologically abnormal (NA). (TIFF) [file pone.0078824.s004.tiff]
