# Supplementary material for: A Machine Learning Approach to Automated Structural Network Analysis: Application to Neonatal Encephalopathy
Source: PLoS One. 2013 Nov 25;8(11):e78824. doi: 10.1371/journal.pone.0078824 (PMC3840059; doi:10.1371/journal.pone.0078824)
Supplement: Table S1 — Clinical measures of neurological outcome. We define classes “−1” (abnormal neurological outcome or NA) and “1” (normal neurological outcome or NN). Class “1” is defined as having one or more of any of the following: NMS>1, seizures, or abnormal neurological evaluation (NE) by a pediatric neurologist at 6 months or 12 months. Missing evaluations at 12 months had not yet been completed. (DOCX) [file pone.0078824.s005.docx]

**Table 1: Clinical measures of neurological outcome**. We define classes “-1” (abnormal neurological outcome or NA) and “1” (normal neurological outcome or NN). Class “1” is defined as having one or more of any of the following: NMS>1, seizures, or abnormal neurological evaluation (NE) by a pediatric neurologist at 6 months or 12 months. Missing evaluations at 12 months had not yet been completed.

| **Subject** | **Gender** | **Sz** | **NMS** | **NE (6 mo)** | **NE (12 mo)** | **Class** |
| --- | --- | --- | --- | --- | --- | --- |
| 1 | M |  | 0 | Normal |  | 1 |
| 2 | F |  | 0 | Normal |  | 1 |
| 3 | M | + | 0 | Normal | Normal | -1 |
| 4 | F |  | 5 | Abnormal | Abnormal | -1 |
| 5 | M |  | 0 | Normal | Unclear | 1 |
| 6 | M |  | 2 | Unclear | Normal | -1 |
| 7 | F | + | 1 | Abnormal | Normal | -1 |
| 8 | M |  | 3 | Abnormal | Normal | -1 |
| 9 | M | + | 0 | Normal | Normal | -1 |
| 10 | F |  | 1 | Unclear | Abnormal | -1 |
| 11 | M |  | 1 | Unclear | Normal | 1 |
| 12 | F |  | 0 | Normal |  | 1 |
| 13 | M |  | 1 | Normal | Normal | 1 |
| 14 | F |  | 1 | Unclear | Normal | 1 |
| 15 | M |  | 0 | Normal | Normal | 1 |
| 16 | M |  | 2 | Unclear | Normal | -1 |
| 17 | F | + | 0 | Normal |  | -1 |
| 18 | F |  | 1 | Normal |  | 1 |
| 19 | F | + | 3 | Abnormal |  | -1 |
| 20 | F |  | 1 | Abnormal |  | -1 |
| 21 | F |  | 0 | Normal |  | 1 |
| 22 | F |  | 0 | Normal |  | 1 |
| 23 | M | + | 2 | Abnormal |  | -1 |
| 24 | F |  | 0 | Normal |  | 1 |
