# Supplementary material for: A Machine Learning Approach to Automated Structural Network Analysis: Application to Neonatal Encephalopathy
Source: PLoS One. 2013 Nov 25;8(11):e78824. doi: 10.1371/journal.pone.0078824 (PMC3840059; doi:10.1371/journal.pone.0078824)
Supplement: Table S2 — Summary of variables used. (DOCX) [file pone.0078824.s006.docx]

**Supplementary Table 2: Summary of variables used**.

|  | **Variable** | **Description** | **Variable** | **Description** |
| --- | --- | --- | --- | --- |
| **NETWORK-RELATED VARIABLES** | *n* | Number of cortical brain regions for parcellation and number of vertices in the network | *n_g_* | Number of vertices in the largest connected component of the network |
|  | *A* | Adjacency matrix | *e_ij_* | Binary edge {0,1} between vertex *i* and *j* |
|  | *c_i_* | Local clustering coefficient of vertex *i* | *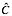* | Average clustering coefficient over all vertices |
|  | *l_ij_* | Geodesic distance between vertex *i* and vertex *j* | *_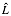_* | Average geodesic length over all vertices |
|  | *t* | Number of 3-cliques in the network | *q* | Number of paths of length 2 in the network |
|  | *T* | Transitivity of the network | *d_i_* | Degree of vertex *i* |
|  | *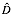* | Average degree over all vertices |  |  |
|  | | | | |
| **PCA-RELATED VARIABLES** | *N* | Number of subjects (24) | *M* | Number of variables measured on the network (149 in the 8-walk subgraph space) |
|  | *X* | Data matrix *(NxM)* | *Z* | Data matrix *(NxM)* with zero mean along each of the *M* dimensions |
|  | *U* | Matrix whose columns are eigenvectors of the covariance matrix *(MxM)* | *W_p_* | Projection of data set onto first *p* eigenvectors of *U (Nxp)* |
|  | | | | |
| **SVM-RELATED VARIABLES** | *x_i_* | Training example *i* (1xM) | *y_i_* | Class label of example *i* {-1, 1} |
|  | *f(x,{w,b})* | Linear separating hyperplane to be learned  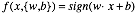 | *w* | Vector of coefficients of the separating hyperplane |
|  | *b* | Bias term of the separating hyperplane | *ξ_i_* | Positive slack variables for each example *i* |
|  | *C* | SVM tuning parameter that controls number of outliers to allow | *L* | SVM Lagrangian |
